# Tracheostomy decannulation protocol in patients with prolonged tracheostomy referred to a rehabilitation hospital: a prospective cohort study

**DOI:** 10.1186/s40560-022-00626-3

**Published:** 2022-07-16

**Authors:** Ting Zhou, Jianjun Wang, Chenxi Zhang, Bin Zhang, Haiming Guo, Bo Yang, Qing Li, Jingyi Ge, Yi Li, Guangyu Niu, Hua Gao, Hongying Jiang

**Affiliations:** Respiratory Rehabilitation Center, Beijing Rehabilitation Hospital of Capital Medical University, Xixiazhuang, Badachu, Shijingshan, Beijing, 100114 China

**Keywords:** Decannulation, Protocol, Rehabilitation, Speaking valve

## Abstract

**Background:**

The aim of the study was to assess the feasibility of a standardized tracheostomy decannulation protocol in patients with prolonged tracheostomy referred to a rehabilitation hospital.

**Methods:**

This prospective cohort study recruited conscious patients with prolonged tracheostomy who were referred to the pulmonary rehabilitation department of a tertiary rehabilitation hospital between January 2019 and December 2021. A pulmonary rehabilitation team used a standardized tracheostomy decannulation protocol developed by the authors. The primary outcome was the success rate of decannulation. Secondary outcomes included decannulation time from referral and reintubation rate after a follow-up of 3 months.

**Results:**

Of the 115 patients referred for weaning from mechanical ventilation and tracheostomy decannulation over the study period, 80.0% (92/115) were finally evaluated for tracheostomy decannulation. The mean time of tracheostomy in patients transferred to our department was 70.6 days. After assessment by a multidisciplinary team, 57 patients met all the decannulation indications and underwent decannulation. Fifty-six cases were successful, and 1 case was intubated again. The median time to decannulation after referral was 42.7 days. Reintubation after a follow-up of 3 months did not occur in any patients.

**Conclusions:**

A standardized tracheostomy decannulation protocol implemented by a pulmonary rehabilitation team is associated with successful tracheostomy decannulation in patients with prolonged tracheostomy. Not every tracheostomy patient must undergo upper airway endoscopy before decannulation. Tolerance of speaking valve continuously for 4 h can be used as an alternative means for tube occlusion. A swallow assessment was used to evaluate the feeding mode and did not affect the final decision to decannulate.

*Trial registration*: 2018bkky-121.

## Background

Major indications for tracheostomy tubes include failure to wean from mechanical ventilation (MV) [1], inability to handle excessive secretions, and upper airway obstruction [2]. Tracheostomy tubes may cause inflammation, stenosis, excessive cough, and impaired swallowing [3, 4]. Once the acute indications for tracheostomy have been resolved, the patients need to be evaluated for safe decannulation. Tracheostomy decannulation can improve patient comfort, physical appearance, swallowing, communication and social inclusion [2, 5]. In addition, it also solves the caregivers' concerns and anxiety about nursing tracheostomy cannula [6]. However, if the decannulation process is performed without correct assessment of pre-decannulation, this can lead to serious complications and even death [7]. There are different decannulation protocols designed mostly for acute intensive care where tracheostomy tubes are expected to be removed after a short time period [6, 8, 9]. There is a lack of information on decannulation concerning post-acute subjects with prolonged tracheostomy. Clinically, many patients often have tracheostomy tubes for a long period of time, especially for patients with difficulty weaning. Therefore, there is a growing need to identify a protocol specifically designed for patients with prolonged tracheostomy to improve the success rate of decannulation in weaning or rehabilitation institutions and reduce the mortality of patients.

In a rehabilitation hospital, every patient with tracheostomy receives clinical rehabilitation fusion management provided by a multidisciplinary pulmonary rehabilitation team. According to evaluation results, they all receive personalized pulmonary rehabilitation treatment, such as airway clearance training, exercise training and respiratory muscle training. The aims of our study were to assess the feasibility of a standardized tracheostomy decannulation protocol implemented by a pulmonary rehabilitation team in patients with prolonged tracheostomy referred to a rehabilitation hospital.

## Methods

Beijing Rehabilitation Hospital of Capital Medical University is a 950-bed tertiary rehabilitation hospital. There are 54 beds in the pulmonary rehabilitation center, including 22 beds in the intensive rehabilitation unit. Weaning from mechanical ventilation and tracheostomy decannulation are two major clinical tasks. The multidisciplinary team of the tracheostomy decannulation team of our department consists of respiratory physicians, critical care physicians, neurologists, physiotherapists, speech therapists, ear, nose, and throat (ENT) specialists, critical care nurses and cardiopulmonary rehabilitation nurses. If the patient has psychological and nutritional problems, we consult and guide the treatment with relevant professional doctors. All tracheostomy patients referred from other general hospitals were assessed by the multidisciplinary pulmonary rehabilitation team with a standardized tracheostomy decannulation protocol.

**Fig. 1:**
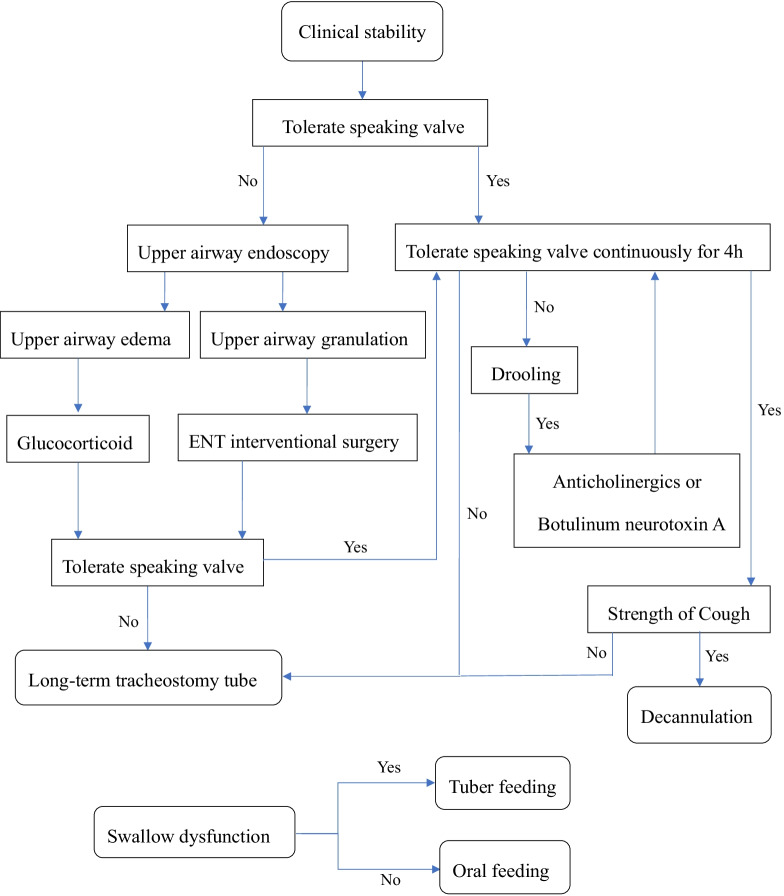
The standardized tracheostomy decannulation protocol

The standardized tracheostomy decannulation protocol is shown in Fig. 1.

Step 1: The patient’s clinical stability was confirmed. The patient (1) was weaned from a ventilator more than 48 h prior; (2) did not have any organ failure; (3) did not have sepsis; (4) had a stable heart rate and blood pressure without the use of vascular active drugs; (5) had any lung infection under control; and (6) had a PaCO_2_ < 60 mmHg.

Step 2: Tolerance to the speaking valve was assessed (Covidien, Italy). The aim of this step was to evaluate the patency of the upper airway. The first wearing time was half an hour, then the blood gas analysis was performed again. If the following conditions occurred, the second step was not passed: (1) dyspnea or exacerbation of dyspnea; (2) severe or persistent cough; (3) decreased oxygen saturation < 90% (permitting supplemental oxygen through the speaking valve’s side span); and (4) blood gas analysis after wearing showed that PH was < 7.35 or c PaCO_2_ was > 60 mmHg. If the speaking valve could not be tolerated, upper airway endoscopy (Olympus fiber bronchoscopy, model BF-P60) was performed. If the upper airway mucosa was congested and edematous, aerosol inhaled through the mouth (not through tracheostomy) of glucocorticoids could be given. If it was very serious, systemic application (intravenous or oral and nasal feeding) could be given. If granulomas and stenosis obstruction of > 50% lumen diameter were present, an ENT specialist assessed the need for local surgery.

Step 3: The wearing time of the speaking valve was extended continuously for 4 h, and no tracheostomy cannula was used for sputum suction within 4 h. When this step failed, the reason perhaps was drooling. Such patients required the administration of anticholinergics or botulinum neurotoxin A to reduce salivary secretion and could finally tolerate the speaking valve continuously for 4 h.

Step 4: Cough strength was also evaluated before decannulation. According to the patient’s consciousness and cognitive level, the modalities of assessment were as follows: clinical assessment, peak cough flow (PCF, Keka, Shanghai) and peak expiratory flow (PEF, Jaeger, MasterScreen, Germany). A PCF > 100 L/min or PEF > 1.67 L/s showed that the patient had good cough ability to decannulate.

Furthermore, assessment of swallowing was also included in the process, but it was used to evaluate the feeding mode of the patient and did not affect the final decision to decannulate. Modalities of assessment were the blue dye test (BDT), fiberoptic endoscopic evaluation of swallowing (FEES) and video fluoroscopic swallowing study (VFSS). If there was swallowing dysfunction, the gastric tube or percutaneous gastrostomy was retained, and the jejunal tube was retained if there was aspiration reflux.

Patients were decannulated when they successfully completed steps 1 through 4. These criteria were adjusted according to the patient's condition and primary diseases. Decannulation failure was defined as the need for reintubation within 48 h after tube removal. Tracheostomy tubes with subglottic suction were used routinely. High-flow oxygen therapy (Airvo 2, Fisher and Paykel Healthcare) was used if secretions were thick and compromised the tube.

### Patients

This prospective cohort study was approved by the ethics committee of Beijing Rehabilitation Hospital of Capital Medical University (2018bkky-121). Informed consent was given by the next of kin, if available, and from the patients upon recovery of competency, in compliance with Chinese law. All patients with prolonged tracheostomy referred for decannulation between January 2019 and September 2021 underwent screening. The exclusion criteria were unconscious patients or death within 2 weeks after referral. The following variables that were recorded at inclusion were demographics, Acute Physiology and Chronic Health Evaluation II (APACHE II) score on admission, primary disease, tracheostomy indication, tracheostomy time before referral, mechanical ventilation time before referral, and hospital length of stay before referral. The following variables were recorded: first time of wearing a speaking valve, time from referral to decannulation, and discharge destination. The primary disease was categorized as lung disease, cardiovascular disease, neuromuscular disease, acute brain injury, thoracoabdominal surgery, cervical spinal cord injury and others. The indications for tracheostomy were categorized as prolonged ventilation, respiratory failure, sputum drainage and difficult airway.

### Endpoints

The primary outcome measured was the success rate of decannulation. Secondary outcomes of interest measured were decannulation time from referral and reintubation after a follow-up of 3 months.

### Statistical analysis

Quantitative parameters are described as the mean (standard deviation, SD), and qualitative parameters are described as numbers (percentages). Analyses were performed using IBM SPSS Statistics version 20.

## Results

**Fig. 2:**
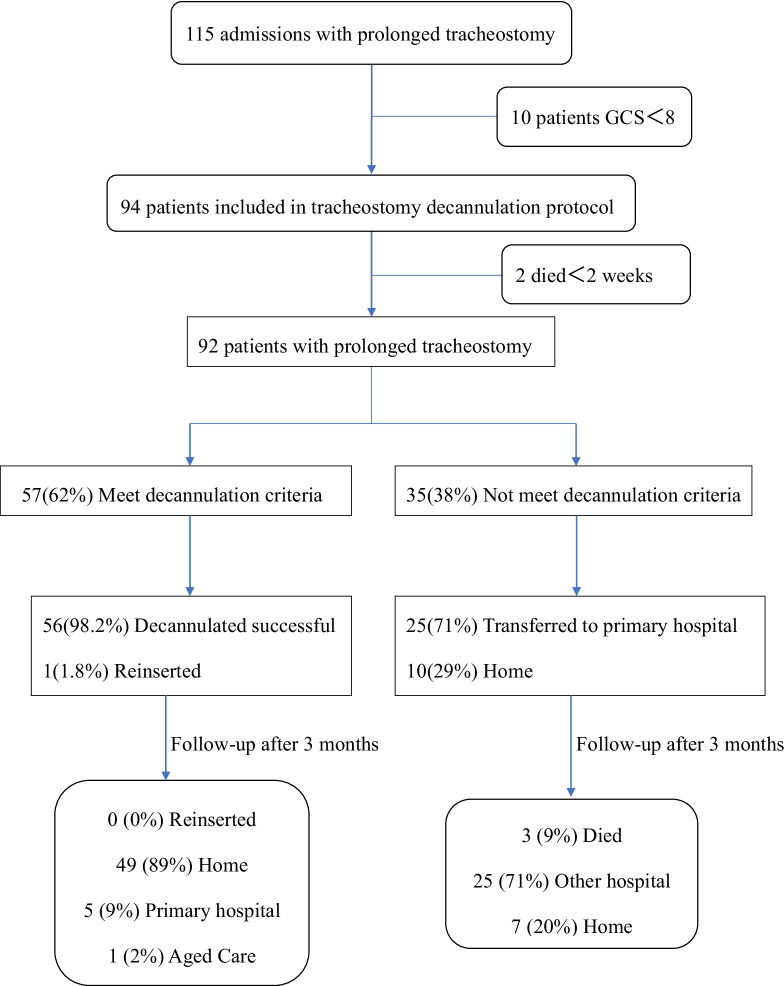
The patient flowchart

Figure 2 shows the patient flowchart. From January 2019 to September 2021, 115 patients with prolonged tracheostomy were referred from general hospitals. Ninety-two were included in the study. The demographic and clinical characteristics of the patients are shown in Table 1.

**Table 1:** The demographic and clinical characteristics of the patients

Patient demographic	*N*
Refer with tracheostomy	92
Age, mean (SD)	64.0 (15.7)
Male, *N*	64 (69.6)
APACHE II score, mean (SD)	12.1 (5.9)
Primary disease	
Lung disease	12 (13.0)
Cardiovascular disease	3 (3.3)
Neuromuscular disease	9 (9.8)
Acute brain injury	37 (40.2)
Thoracoabdominal surgery	11 (12.0)
Cervical spinal cord injury	17 (18.5)
Others	3 (3.3%)
Tracheostomy indication	
Prolonged ventilation	17 (18.5)
Respiratory failure	38 (41.3)
Sputum drainage	31 (33.7)
Difficult airway	6 (6.5)
Tracheostomy time before refer (days)	70.6 (74.8)
Mechanical ventilation time before refer (days)	37.2 (50.4)
Hospital stay before refer (days)	89.0 (83.8)
First speaking valve after refer (days)	17.4 (10.5)
Decannulated (successful/decannulated)	56/57
Not decannulated	35 (38.0)
Time to decannulation after refer (days)	42.7 (18.3)
Reintubation after follow-up of 3 months	0

Data are presented as number (percentage) or mean (standard deviation). Hyphen indicates data not available

*APACHE* Acute Physiology and Chronic Health Evaluation, *SD* standard deviation

### Primary outcome

Of the 92 patients, 57 patients (62.0%) met the criteria of the decannulation protocol after assessment by a multidisciplinary rehabilitation team, 56 (98.2%) were decannulated successfully prior to discharge, and only one patient had a tracheostomy inserted 10 h after decannulation. He was a successful businessman, but a sudden car accident made him a patient with a high cervical spinal cord injury (C3 ASIA-A). Severe pneumonia occurred after the injury, and tracheotomy and invasive ventilator assisted ventilation were performed. This misfortune left the patient with severe psychological problems. One month after being referred to our hospital, the patient passed the protocol and was performed decannulation. Within 9 h after decannulation, the patient's vital signs did not change compared with pre-decannulation. But after 9 h, the patient experienced decreased oxygen saturation to 88%. Nurses immediately performed sputum suction, and oxygen saturation could recovered to 95%. However, the patient was already in a state of extreme fear and strongly demanded reintubation. Later, he turned to a psychiatrist for psychotherapy and drug treatment and was ready to try decannulation again. However, the patient was transferred to a local hospital due to financial problems. Therefore, the patient was lost to follow-up.

Of the 35 patients who did not meet the protocol criteria, eight patients stopped at step 1 due to progression of the primary disease, ten patients (three patients had granulation tissue hyperplasia, one patient had fibrous scarring, six patients had vocal cord paralysis) stopped at step 2 due to the obstructed upper airway, three patients stopped at step 3 due to untreatable drooling, 14 patients stopped at step 4 due to cough strength.

### Secondary outcomes

The mean time from referral to decannulation was 42.7 days, and reintubation after a follow-up of 3 months did not occur in any cases. The decannulation time for patients with lung disease was 41.8 ± 21.4 days, neuromuscular disease was 66.5 ± 31.0 days, acute brain injury was 47.3 ± 14.0 days, thoracoabdominal surgery was 32.9 ± 12.8 days, and cervical spinal cord injury was 33.6 ± 9.4 days. After decannulation, 49 patients (89%) were discharged home, 5 (9%) were discharged to a primary hospital, and 1 (2%) was discharged to aged care. Of the 35 patients (38%) not decannulated and a follow-up of 3 months, 3 (9%) died and 25 (71%) were discharged to a rehabilitation unit or another hospital, and 7 (20%) were discharged home. Twenty-two patients were unable to tolerate the speaking valve. All of them underwent fiberoptic bronchoscopy to assess the patency of the upper airway. Nine patients had upper airway edema, and after adding local and systemic hormone therapy, all tolerated the speaking valve. There were 6 cases of granulation tissue hyperplasia, of which 1 case was tracheal mucosal trauma caused by the end of the tracheostomy tube, which did not prevent decannulation. Five cases exceeded 50% of the tracheal diameter and were transferred to a specialized hospital for ENT interventional surgery. Two patients replaced the extended tracheostomy tube and decannulated successfully after the multidisciplinary team assessed that all the protocol criteria were met. One patient with a case of fibrous scarring was transferred to a specialized hospital for interventional surgery. Six patients had vocal cord paralysis and did not tolerate the speaking valve and long-term indwelling tracheostomy tube.

Ten patients tolerated the speaking valve but could not tolerate the speaking valve continuously for 4 h because of drooling. Such patients required the administration of anticholinergics (scopolamine hydrobromide patch) or botulinum neurotoxin A to reduce salivary secretion. Ten patients all used a scopolamine hydrobromide patch, and 5 patients could tolerate the speaking valve continuously for 4 h after therapy. The other 5 patients needed to be injected with botulinum neurotoxin A, and 2 patients succeeded in tolerating the speaking valve continuously for 4 h. The treatment of 3 patients was not effective, and they could not tolerate the speaking valve continuously for 4 h.

## Discussion

Prolonged tracheostomy may induce many late complications, including aggravation of dysphagia, bleeding, tracheal stenosis, granuloma, psychosocial side effects, etc. [3, 4]. There is broad variability in tracheostomy care and decannulation practices and a lack of a universally accepted decannulation protocol, especially for prolonged tracheostomy patients [10]. The present study investigated the success of decannulation in patients with prolonged tracheostomy referred to a rehabilitation hospital. According to the data of 3 years, after the evaluation of the multidisciplinary team, a total of 57/92 patients met the protocol criteria and underwent decannulation. The success rate of decannulation was 98.2%; only 1 patient needed recannulation, and the failure rate was 1.8%. The incidence of recannulation was lower than that in other studies (2–5%) [11], and no patient was intubated after 3 months of follow-up after decannulation. One reason was that the criteria for the decannulation protocol were very strict. After assessment, only 62.0% of patients met the criteria, which was lower than in the study by Pandian V [12], in which the decannulation rate was as high as 88%.

Because the patients referred to our department for tracheostomy decannulation had a long tracheostomy time, the mean time was 70.6 days, and the mean time of mechanical ventilation was 37.2 days, which was longer than any other study (days) [13, 14]. The evaluation of the upper airway is more important because there are many problems with upper airway patency in patients with long-term tracheostomy [5]. Clinicians evaluated airway patency directly by visualization through endoscopy or assumed a patent airway when a patient tolerated physiological decannulation, which was assessed by occlusion [9], a one-way speech value [15], or downsizing to smaller tubes [16]. Some researchers suggested upper airway endoscopy for all patients before tracheostomy decannulation [17]. However, the others did not use bronchoscopy or upper airway endoscopy, yet they successfully decannulated 60% of the patients [9]. Antony E Tobin et al. [15] mentioned in their study the use of the Passy-Muir valve to assess upper airway patency, but there are no details on how to use it, except in some patients still using tracheostomy tube occlusion. We routinely used a speaking valve instead of occlusion to determine the patency of the upper airway. The speaking valve is unidirectional, allowing air to be drawn through the tracheostomy tube during inspiration (with small inspiratory pressure) and has an automatic closure mechanism that directs airflow into the upper airway during the expiratory phase. When using a speaking valve, airflow changes, so the explanation of the process and how the valve works to the patient assists with reducing anxiety and encouraging normal breathing patterns. Guerlain et al. [18] considered fiberoptic endoscopy only in cases of repeated failed decannulation. We prescribed fiberoptic endoscopy only when the patient could not tolerate the deflation of the cuff and the use of a speaking valve. In our study, 70 patients passed the upper airway patency evaluation because they could tolerate the speaking valve. These patients did not undergo laryngoscopy, and 41 were successfully decannulated. One study reported that 67% of patients with tracheostomies were found to have airway abnormalities during airway endoscopy. Findings included tracheal granulomas, tracheomalacia, tracheal stenosis, and vocal cord dysfunction [19]. Some of the abnormalities visualized, such as minor mucosal trauma from the tracheostomy tube or suction catheter, may not be clinically important and usually do not prevent decannulation. It has been demonstrated that patients who successfully pass a tracheostomy tube occlusion protocol can be safely decannulated without first undergoing fiberoptic bronchoscopy [20]. One innovation of our study is that tolerance of tube occlusion was not used as the standard to be met in the decannulation protocol for any of the patients. Many studies state that the ability to tolerate tracheostomy tube occlusion is an important factor to ensure readiness prior to a patient undergoing decannulation [7, 12, 21–23]. It is common practice to cap the tracheostomy tube for 24 h to see whether the patients can breathe on their own [12]. However, occluding the tube leads to increased airway resistance, resulting in increased respiratory work in patients with marginal ventilation [24]. Moreover, this protocol may delay decannulation because some patients who do not meet the occlusion criteria may still be able to undergo decannulation successfully [25]. In addition, one study suggested that the failure of capping trials could lead to a sequence of clinical deterioration [12, 25]. Patients did not undergo capping trials, and the decision to decannulate was based on tolerance of the speaking valve continuously for 4 h. In addition, this criterion could evaluate secretion at the same time, which has also been reported in many studies. Decannulation failures could contribute to uncontrolled secretions and severe glottic stenosis. Pandian et al. [12] considered suctioning less frequently than every 4 h as a standardized tracheostomy decannulation protocol in patients with mixed abnormalities. For patients with acquired brain injury, some authors considered two suctions or less every 8 h as criteria [21]. Based on the clinical practice of our department, tolerance of a speaking valve continuously for 4 h instead of tube occlusion was proposed. This criterion may make it possible for patients with extremely poor pulmonary capacity and who are unable to tolerate tube occlusion to decannulate successfully. The cuff is the crucial interface between the tube and the trachea that may prevent leakage of secretions that lead to tracheal ischesis [26]. When patients with drooling used a speaking valve, cuff deflation would result in a large amount of saliva flowing into the airway, finally appearing as dyspnea, persistent cough, decreased oxygen saturation and so on. Such patients could not tolerate the speaking valve continuously for 4 h, required the administration of anticholinergics or botulinum neurotoxin A to reduce salivary secretion, and could then finally tolerate the speaking valve continuously for 4 h.

Assessment of cough strength is an important criterion for considering tracheostomy decannulation. Bach and Saporito [27] found that only the ability to generate a PCF of at least 160 L/min predicted the success of decannulation. In our study, the mean PEF of patients whose cough strength was enough to clear sputum was 107.7 L/min. Therefore, we conjectured that a PCF or PEF of at least 100 L/min would predict the effectiveness of coughing. The results confirmed that the patients (PCF/PEF > 100 L/min) were successfully decannulated, and reintubation after a follow-up of 3 months did not occur.

Many studies confirmed that failing a swallow examination predicted failure of decannulation [28]. However, we found that as long as patients can effectively manage oral secretions and have the ability to cough, even if they have swallowing dysfunction, they can still decannulate successfully and nasogastric tube, jejunal tube or percutaneous gastrostomy can remain. In addition, because tracheostomy may increase the risk of aspiration as it interferes directly in the pharyngeal phase of swallowing, the failure of a swallowing evaluation does not indicate that the tube cannot be decannulated [29]. In this study, 29 patients could not pass the swallowing evaluation, but after the evaluation, the tracheostomy cannula was successfully decannulated, the gastric tube was retained in 23 cases, the jejunal tube was retained in 3 cases, and gastrostomy was retained in 3 cases and removed in 3 cases after swallowing function was recovered.

Due to the limited medical resources in the intensive care unit, many patients with tracheostomy are transferred to a weaning facility, such as a step-down unit or a long-term care hospital. However, it was reported that ICU patients who received tracheostomies and were sent to the ward had significantly higher odds of death than those patients decannulated in the ICU prior to discharge. Many systematic reviews also pointed out that the multidisciplinary tracheostomy team is the most appropriate team for tracheostomy management and is associated with a shorter time from the ICU to the general ward and tracheostomy decannulation for patients [15]. Ilaria Zivi et al. [30] stated that an early neurorehabilitation protocol helps to reduce the time to decannulation in tracheostomized patients with severe acquired brain injury. Most multidisciplinary research teams include ICU physicians, otolaryngologists and respiratory physicians [12]. As in Antony E’s study, physiotherapists were included in our multidisciplinary team [15]. In weaning facilities, a multidisciplinary team manages medical care and rehabilitation, which is conducive to weaning from ventilation and tracheostomy decannulation. All patients referred to undergo tracheostomy decannulation in our center were evaluated by our multidisciplinary rehabilitation team, and a pulmonary rehabilitation program was started after assessment. As long as the patient was clinically stable, they routinely started personalized pulmonary rehabilitation tailored to their assessment. Because of long-term tracheostomy, many patients could not master the physiological way of breathing from the upper airway when deflating the cuff, especially when wearing the speaking valve. The physiotherapist could help moderate the breathing mode and gradually prolong the wearing time. When the patients had many secretions, physiotherapists used a variety of airway clearance techniques to help the patients remove the secretions in the trachea, which was helpful for decannulation. If the strength of cough was weak, physiotherapists could increase cough training and mechanical inhalation and exhalation. Successful implementation of a rehabilitation and weaning protocol is dependent on careful planning and detailed communication between the rehabilitation specialist and clinicians during the pulmonary rehabilitation process.

### Study limitations

The main limitation of this study was the prospective design in a single center. There was no randomized control method to confirm noninferiority of speaking valves to tube occlusion and to clarify the role of pulmonary rehabilitation in tracheostomy decannulation.

## Conclusions

A standardized decannulation protocol implemented by a multidisciplinary rehabilitation team was associated with successful tracheostomy decannulation in patients with prolonged tracheostomy. Evaluation of patency of the upper airway is very important for patients with prolonged tracheostomy. However, not every patient needs to be evaluated by endoscopy. Tolerance of a speaking valve continuously for 4 h was used as an alternative means for tube occlusion. A swallow assessment was used to evaluate the feeding mode and did not affect the final decision to decannulate.

## Data Availability

The datasets used and/or analyzed during the current study are available from the corresponding author on reasonable request.

## Abbreviations

MV: Mechanical ventilation
ENT: Ear, nose, and throat
BDT: Blue dye test
FEES: Fiberoptic endoscopic evaluation of swallowing
VFSS: Video fluoroscopic swallowing study
APACHE II: Acute Physiology and Chronic Health Evaluation II
